# Starvation-Induced Liver Injury During the Early Phase of Refeeding in a Patient With Anorexia Nervosa

**DOI:** 10.7759/cureus.95513

**Published:** 2025-10-27

**Authors:** Kazuhide Takata, Keisuke Matsumoto, Takashi Tanaka, Keiji Yokoyama, Fumihito Hirai

**Affiliations:** 1 Department of Gastroenterology and Medicine, Fukuoka University Faculty of Medicine, Fukuoka, JPN

**Keywords:** anorexia nervosa, body weight, liver injury, refeeding, starvation-induced autophagy

## Abstract

We report a case of a 41-year-old woman with longstanding anorexia nervosa (AN) who developed marked elevation in transaminase levels during early refeeding with enteral nutrition. Imaging and lab studies excluded viral hepatitis, ischemic injury, and refeeding syndrome. Her liver enzymes rapidly normalized with continued caloric intake and weight gain. This case supports the concept of starvation-induced liver injury, possibly due to autophagy, and highlights the importance of distinguishing it from refeeding syndrome, as treatment strategies differ significantly. Body weight trends may aid in predicting the trajectory of liver injury during nutritional rehabilitation in AN.

## Introduction

Anorexia nervosa (AN) is a psychiatric eating disorder characterized by a restrictive caloric intake relative to the body’s needs, leading to significant weight loss and damage to multiple organ systems in severe cases. The clinical course of liver injury associated with AN remains poorly understood [1,2]. In particular, abnormal liver enzyme elevation may occur during the refeeding phase, raising concern for refeeding syndrome, a potentially fatal condition marked by electrolyte shifts, especially hypophosphatemia, due to rapid metabolic transition following nutritional rehabilitation. However, recent literature has also described a distinct entity known as starvation-induced liver injury, characterized by marked transaminase elevation in the absence of fatty liver or hepatomegaly, likely mediated by autophagy-related hepatocyte membrane changes. Here, we report a case of a patient with severe AN who developed acute liver enzyme elevation during early refeeding. This case highlights the importance of differentiating between refeeding syndrome and starvation-induced liver injury, as their management strategies diverge significantly.

## Case presentation

A 41-year-old woman with a 20-year history of AN was admitted to our hospital for ongoing management of AN-related weight loss, following a 10-day hospitalization at another facility. She had no significant medical history other than AN, no history of alcohol consumption, and was not taking any medications. Her vital signs were largely stable, except for mild hypotension (94/57 mmHg). The patient had a critically low body weight (height: 149 cm; weight: 22.7 kg; body mass index (BMI): 10.2 kg/m2). Laboratory findings revealed elevated liver enzymes (aspartate aminotransferase (AST): 73 U/L; alanine aminotransferase (ALT): 141 U/L); hypoalbuminemia (3.8 g/dL); and hypoglycemia (62 mg/dL).

**Figure 1:**
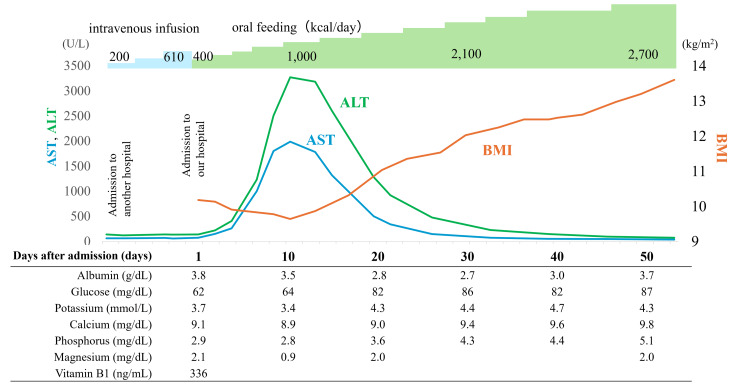
Clinical course of liver enzyme levels and body weight AST: aspartate aminotransferase; ALT: alanine aminotransferase; BMI: body mass index After oral nutrition was started, the liver damage rapidly worsened, but then rapidly improved with weight gain

The patient was transitioned from intravenous therapy, which had been administered at the previous facility, to enteral nutritional therapy via a nasogastric tube. The initial caloric intake was set at 400 kcal/day, divided into two doses at a rate of 100 kcal/h. This intake was gradually increased by 200 kcal every 2-3 days, with close monitoring for signs of refeeding syndrome. A rapid increase in transaminase levels was observed four days after the initiation of enteral nutrition (Figure 1, Table 1).

**Table 1:** Structured timeline of nutritional intervention and hepatic response Summary of the patient’s clinical course across three phases: (i) pre-refeeding, (ii) early refeeding, and (iii) nutritional recovery. The table outlines the timing of refeeding initiation, changes in body weight, and transaminase levels in relation to nutritional status

Phase	Days after admission	Refeeding	Body weight	Transaminase levels
(i) Pre-refeeding	Day 0	Not started	↓	Mild elevation
		Infusion only		
(ii) Early refeeding	Day 1-10	Started	↓	Marked elevation
		Gradual caloric increase		
(iii) Nutritional recovery	Day 11-	Ongoing	↑	Rapid normalization
		Gradual caloric increase		

**Figure 2:**
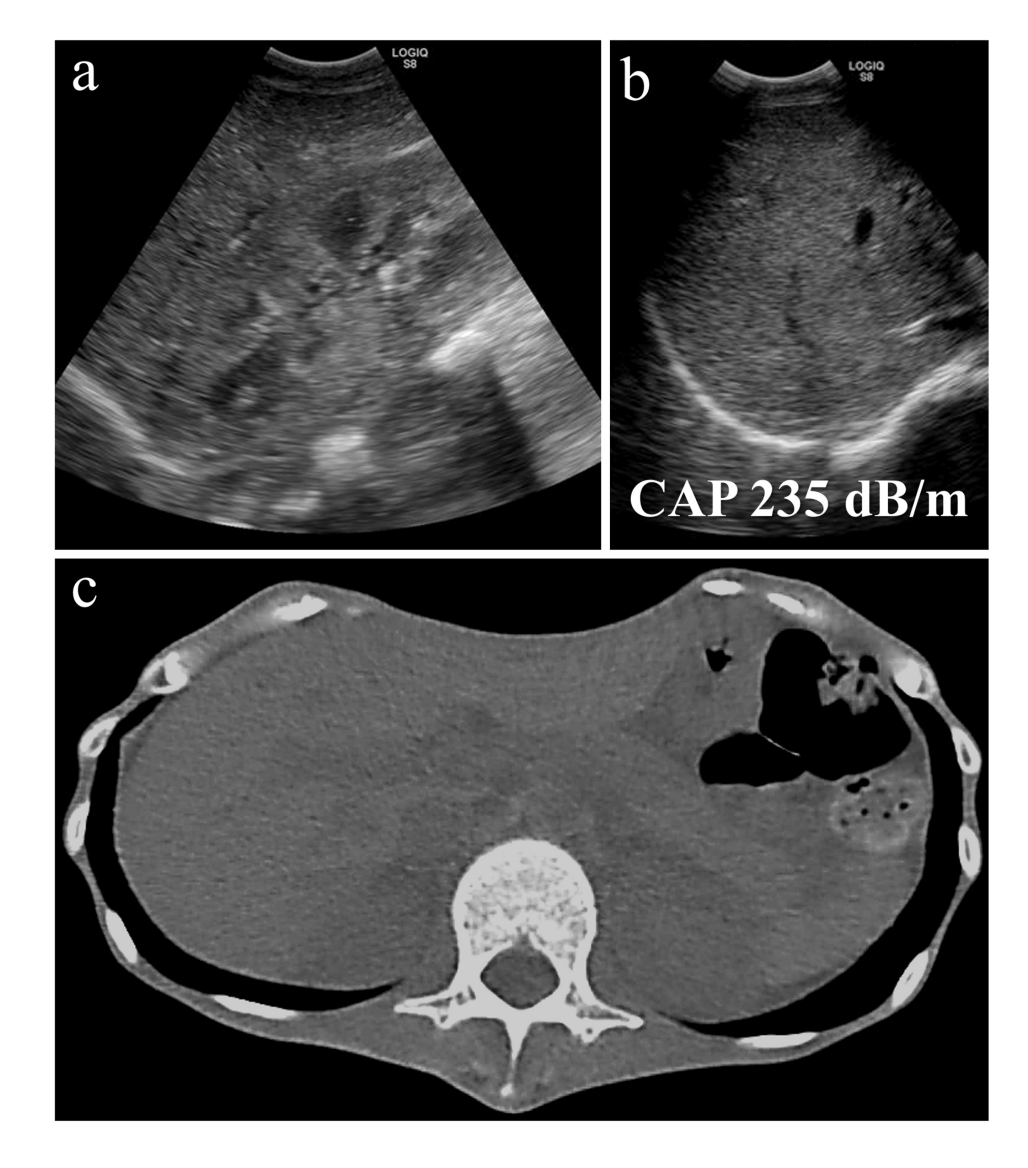
Abdominal imaging during the phase of rapidly elevated transaminases CAP: controlled attenuation parameter Abdominal imaging during the phase of rapidly elevated transaminases. (a) Abdominal ultrasonography did not show hepatorenal echo contrast. (b) Deep echo attenuation was not observed. (c) Abdominal computed tomography did not show hepatomegaly

No significant hypophosphatemia or hypokalemia was observed. Serologic testing for hepatitis A, B, C, and E was negative. Imaging studies, including abdominal ultrasound and computed tomography (CT), showed no evidence of hepatomegaly, fatty liver, or obstruction of the hepatic blood vessels (Figures 2a-2c).

These findings suggested that acute viral hepatitis and ischemic liver injury were unlikely. Refeeding syndrome was also excluded based on the absence of hallmark laboratory abnormalities such as hypophosphatemia and hypokalemia, as well as the lack of clinical signs, including fluid overload, cardiac arrhythmia, or neuromuscular symptoms. Imaging studies revealed no evidence of hepatomegaly or hepatic steatosis, which are sometimes observed in refeeding syndrome. Combined with the absence of hallmark electrolyte disturbances and clinical signs, these findings supported the exclusion of refeeding syndrome. Nutritional therapy was continued, and enteral feeding via nasogastric tube was transitioned to oral feeding. Ten days after the initiation of enteral nutrition, the patient exhibited weight gain alongside a rapid improvement in liver enzyme levels (Figure 1, Table 1).

## Discussion

This case underscores the clinical significance of the following observations: (i) profound weight loss accompanied by mild liver injury prior to refeeding; (ii) worsening liver injury during the early phase of refeeding; and (iii) rapid normalization of liver enzymes with continued feeding and weight gain (Table 1).

Liver injury occurs in approximately 30-60% of patients with AN [1,2]. Hanachi et al. reported transaminase elevation in 43% of patients with AN upon admission [2]. In this case, mild transaminase elevation was noted at admission but worsened significantly during the early days of refeeding. Similar cases have been previously reported [3-10]. Imaeda et al. found that 45.8% of AN patients with normal ALT at admission developed elevated ALT after refeeding [3].

Liver injury during renutrition in patients with AN includes multiple pathologies, including (i) ischemic hepatitis due to low blood flow, (ii) liver injury due to refeeding syndrome, and (iii) starvation-induced liver injury [1,9-12]. The patient had mild hypotension but maintained hemodynamic stability, ruling out ischemic hepatitis. Refeeding syndrome is a life-threatening disorder characterized by electrolyte shifts, especially hypophosphatemia, following nutritional rehabilitation. In this case, however, hallmark features of refeeding syndrome were absent: serum phosphate levels remained within normal limits, and no hypokalemia, fluid overload, or cardiac arrhythmia were observed. Imaging showed no hepatic enlargement or steatosis, and transaminase levels were markedly elevated, well beyond the mild elevations typically seen in refeeding syndrome [11]. These findings, along with stable hemodynamics, supported the exclusion of refeeding syndrome as the cause of liver injury.

Another hepatic complication to be aware of in AN is starvation-induced liver injury [1,4-7,12]. Unlike refeeding syndrome, starvation-induced liver injury is characterized by a significant increase in serum transaminase levels and the absence of fatty liver and hepatomegaly, consistent with our case. The most important point is that the therapeutic interventions for refeeding syndrome and starvation-induced liver damage are completely different. In refeeding syndrome, it is necessary to reduce calorie intake, especially at the start of nutritional therapy. On the other hand, in starvation-induced liver damage, it is necessary to increase calorie intake and body weight [3,5,8]. The mechanism is not fully understood, but it is proposed to be caused by starvation-induced autophagy [6,8,13,14]. This process, triggered by nutritional stress due to nutrient deficiency, leads to increased permeability of the hepatocyte plasma membrane without inducing apoptotic processes, thus causing severe transaminase elevation due to the release of aminotransferases into the blood without resulting in hepatocyte death [13]. Previous reports suggest that the initial 5-7 days of refeeding in patients with AN constitute the "phase of stabilization" during which a transition from a catabolic to an anabolic state occurs [15]. This metabolic shift may place a strain on the entire body, including the liver, and may induce autophagy.

Since refeeding syndrome was ruled out in this case, the patient continued to increase her caloric intake. Weight gain through continued nutritional therapy likely suppressed autophagy, leading to rapid improvement in liver function. These findings align with previous reports [6,15,16]. Cuntz et al. demonstrated that body weight strongly correlates with transaminase levels, and nutritional rehabilitation can quickly normalize hypertransaminasemia [16].

This case report has several important limitations that need to be considered. First, because of its invasive nature, a liver biopsy was not performed, and therefore, histological examination was not conducted. However, this case was managed without any invasive procedures, relying on appropriate blood sampling, imaging findings, and the clinical course. Second, the initial calorie intake during renutrition therapy may have been low. Because this patient was at high risk for developing refeeding syndrome, we started with a low-calorie diet and slowly increased the intake according to the NICE guidelines [17]. However, recent literature suggests that short-term efficacy and safety may be achieved even with a higher initial caloric intake in severely malnourished patients, provided that careful monitoring is ensured [18-20]. These studies propose that early aggressive nutrition may suppress starvation-induced autophagy more rapidly, potentially mitigating liver injury. While our conservative approach prioritized safety, future protocols may benefit from individualized strategies that balance the risk of refeeding syndrome with the need to reverse catabolic stress efficiently.

## Conclusions

Patients with AN may develop transient liver injury during refeeding, which often improves rapidly with continued nutritional therapy, provided that refeeding syndrome has been effectively excluded. Trends in body weight could serve as a useful predictor for the trajectory of liver injury.

## References

[1] Rosen E, Bakshi N, Watters A, Rosen HR, Mehler PS (2017). Hepatic complications of anorexia nervosa. Dig Dis Sci.

[2] Hanachi M, Melchior JC, Crenn P (2013). Hypertransaminasemia in severely malnourished adult anorexia nervosa patients: risk factors and evolution under enteral nutrition. Clin Nutr.

[3] Imaeda M, Tanaka S, Fujishiro H (2016). Risk factors for elevated liver enzymes during refeeding of severely malnourished patients with eating disorders: a retrospective cohort study. J Eat Disord.

[4] Nadelson AC, Babatunde VD, Yee EU, Patwardhan VR (2017). Expanding the differential diagnosis for transaminitis in patients with anorexia nervosa. J Gen Intern Med.

[5] Tsutsumi M, Okamoto N, Tesen H, Kijima R, Yoshimura R (2024). Choosing appropriate nutritional therapy for patients with anorexia nervosa exhibiting liver dysfunction: a case report. Cureus.

[6] Pinheiro J, Jameel I, Palejwala A (2021). Deranged liver function tests and liver insults in malnourished patients: a report of two cases and literature review. Cureus.

[7] Su A, Choe M, Birkness JE, Limketkai B, Chen PH (2021). Two acute liver injuries in a patient with malnutrition. J Med Cases.

[8] Narayanan V, Gaudiani JL, Harris RH, Mehler PS (2010). Liver function test abnormalities in anorexia nervosa-cause or effect. Int J Eat Disord.

[9] Giordano F, Arnone S, Santeusanio F, Pampanelli S (2010). Brief elevation of hepatic enzymes due to liver ischemia in anorexia nervosa. Eat Weight Disord.

[10] Tomita K, Haga H, Ishii G (2014). Clinical manifestations of liver injury in patients with anorexia nervosa. Hepatol Res.

[11] Heuft L, Voigt J, Selig L, Stumvoll M, Schlögl H, Kaiser T (2023). Refeeding syndrome. Dtsch Arztebl Int.

[12] Biolato M, Terranova R, Policola C, Pontecorvi A, Gasbarrini A, Grieco A (2024). Starvation hepatitis and refeeding-induced hepatitis: mechanism, diagnosis, and treatment. Gastroenterol Rep (Oxf).

[13] Rautou PE, Cazals-Hatem D, Moreau R (2008). Acute liver cell damage in patients with anorexia nervosa: a possible role of starvation-induced hepatocyte autophagy. Gastroenterology.

[14] Schuster K, Staffeld A, Zimmermann A, Böge N, Lang S, Kuhla A, Frintrop L (2024). Starvation in mice induces liver damage associated with autophagy. Nutrients.

[15] Golden NH, Meyer W (2004). Nutritional rehabilitation of anorexia nervosa. Goals and dangers. Int J Adolesc Med Health.

[16] Cuntz U, Voderholzer U (2022). Liver damage is related to the degree of being underweight in anorexia nervosa and improves rapidly with weight gain. Nutrients.

[17] (2020). Eating disorders: recognition and treatment. https://www.nice.org.uk/guidance/ng69.

[18] Academy for Eating Disorders' Medical Care Standards Committee (2021). Academy for Eating Disorders' Medical Care Standards Committee. Eating disorders: a guide to medical care. Eating Disorders: A Guide to Medical Care.

[19] Garber AK, Cheng J, Accurso EC (2021). Short-term outcomes of the study of refeeding to optimize inpatient gains for patients with anorexia nervosa: a multicenter randomized clinical trial. JAMA Pediatr.

[20] Roman C, Aglave R, Farine S, Joris C, Lefebvre L, Vermeulen F (2024). High-calorie refeeding in adolescents with anorexia nervosa: a narrative review. Acta Gastroenterol Belg.

